# Correlation between Focal Nodular Low Signal Changes in Hoffa's Fat Pad Adjacent to Anterior Femoral Cartilage and Focal Cartilage Defect Underlying This Region and Its Possible Implication

**DOI:** 10.1155/2016/8675160

**Published:** 2016-04-27

**Authors:** Chermaine Deepa Antony, John George, Wuey Min Ng, Manimalar Selvi Naicker Subramaniam

**Affiliations:** ^1^University of Malaya Research Imaging Centre, Faculty of Medicine, University of Malaya, 59100 Kuala Lumpur, Malaysia; ^2^Department of Orthopaedic Surgery, Faculty of Medicine, University of Malaya, 59100 Kuala Lumpur, Malaysia; ^3^Department of Pathology, Faculty of Medicine, University of Malaya, 59100 Kuala Lumpur, Malaysia

## Abstract

*Purpose*. This study investigates the association between focal nodular mass with low signal in Hoffa's fat pad adjacent to anterior femoral cartilage of the knee (FNMHF) and focal cartilage abnormality in this region.* Method*. The magnetic resonance fast imaging employing steady-state acquisition sequence (MR FIESTA) sagittal and axial images of the B1 and C1 region (described later) of 148 patients were independently evaluated by two reviewers and categorized into four categories: normal, FNMHF with underlying focal cartilage abnormality, FNMHF with normal cartilage, and cartilage abnormality with no FNMHF.* Results*. There was a significant association (*p* = 0.00) between FNMHF and immediate adjacent focal cartilage abnormality with high interobserver agreement. The absence of focal nodular lesions next to the anterior femoral cartilage has a very high negative predictive value for chondral injury (97.8%). Synovial biopsy of focal nodular lesion done during arthroscopy revealed some fibrocollagenous tissue and no inflammatory cells.* Conclusion*. We postulate that the FNMHF adjacent to the cartilage defects is a form of normal healing response to the cartilage damage. One patient with FHMHF and underlying cartilage abnormality was rescanned six months later. In this patient, the FNMHF disappeared and normal cartilage was observed in the adjacent region which may support this theory.

## 1. Introduction

Chondral and osteochondral injuries of the knee are common. This has been documented in magnetic resonance imaging (MRI) studies [1], in studies of asymptomatic athletes [2], and in patients undergoing knee arthroscopy [3, 4]. These injuries are often seen in young and active individuals, and unfortunately, the joint cartilage has limited capacity for repair. Many peer review articles have demonstrated comparable diagnostic accuracy of MRI to conventional arthroscopy in detecting chondral abnormalities in the knee [5, 6]. Although the presence of concomitant medial meniscus tear and anterior crucial ligament injury on MRI may alert the radiologist to search for possible chondral abnormalities, these do not localize them [7]. There are also articles that have demonstrated the role of synovium in tissue repair [8–10]. To this end, we proposed that the presence of focal nodular mass with low signal in Hoffa's fat pad adjacent to anterior femoral cartilage of the knee (FNMHF) is strongly associated with underlying cartilage abnormalities as a form of normal healing response. If this is substantiated in our study, this would no doubt help physicians to predict cartilage damage and discourage surgeons from shaving off any reactive synovial changes adjacent to the anterior femoral condyle during arthroscopy which may contribute to the healing process. Furthermore, the absence of FNMHF on MRI would indicate a low likelihood of an acute chondral injury.

## 2. Material and Methods

This is a prospective study to investigate if there is a relationship between FNMFH and cartilage defects underlying this region. Approval was obtained from the Medical Ethics Committee at the University Malaya Medical Centre in 2013.

### 2.1. Inclusion and Exclusion Criteria

Patients between the ages of 15 to 50 years who underwent MRI of the knee between May 2012 and June 2014 at the University Malaya Medical Centre were included in this study. Patients with a history of recent trauma, surgery, or joint prosthesis; recent arthroscopy less than 6 months prior to MRI; intra-articular or systemic corticosteroid use less than 3 months before MRI; septic arthritis; medial plicae; inflammatory arthritis; and more than 3 mm generalized synovitis in the superior patellar recess of the knee suggesting presence of generalized synovitis were excluded from this study.

### 2.2. Patient Preparation

A patient was not required to fast unless sedation was required. Patients were told to remove all metallic and electronic objects before entering the scan room.

### 2.3. MRI Study Protocol/Sequences

Scans were performed on clinical 3.0 T Sigma® Hex MR Systems (GE Healthcare, Milwaukee, Wisconsin, USA). A routine MR knee protocol that included sagittal 3D fast imaging employing steady-state acquisition (FIESTA) (TE/TR 3.0/6.455 ms) sagittal proton density weighted (PDW) (TE/TR) and axial 3D FIESTA (TE/TR 3.008/6.427 ms) images was used. Section thickness was 2.0 mm, intersection gap was 1.0 mm, field of view was 18 × 18 cm, and the matrix was 384 × 256 pixels.

### 2.4. Data Collection and Storage

MRI images were transferred to General Electric workstations for reporting, processing, measurements, and analysis. The images were stored on the picture archiving and communication archiving system.

**Figure 1:**
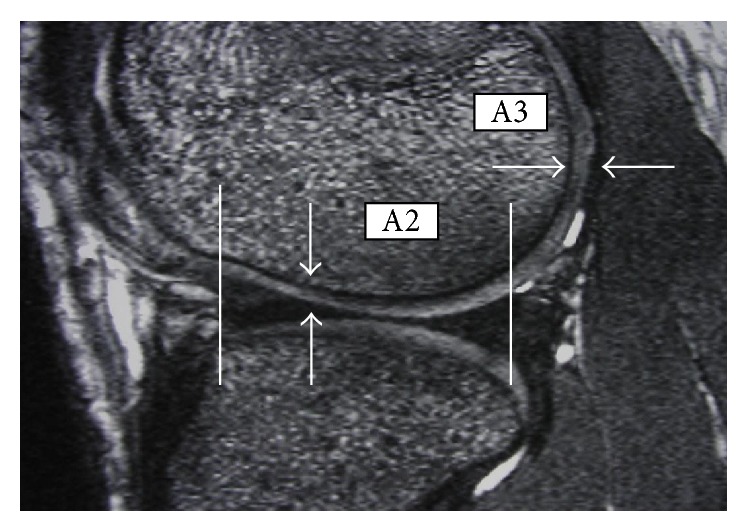
Mapping of MRI showing regions that are A2 and A3. White arrows show thickness in these 2 regions.

**Figure 2:**
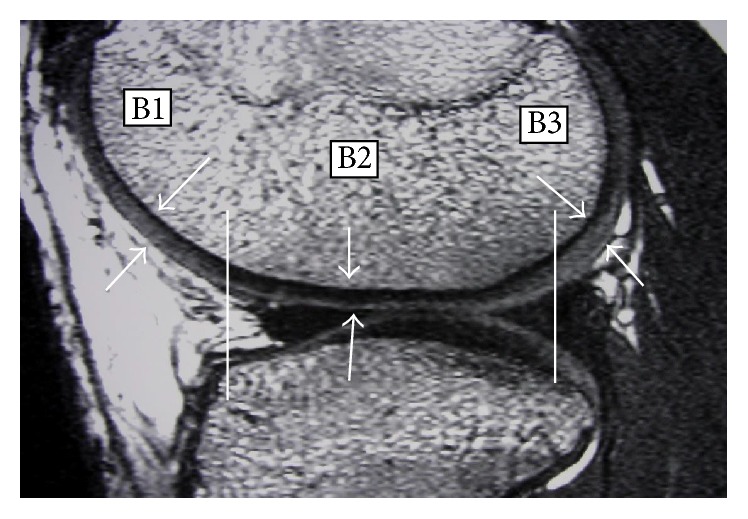
Mapping of MRI showing regions that are B1, B2, and B3. White arrows show cartilage thickness in these three regions.

**Figure 3:**
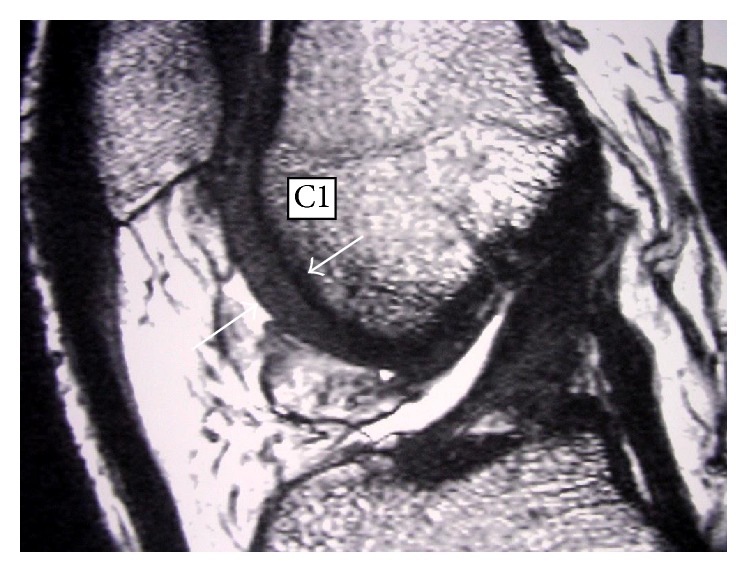
Mapping of MRI showing region that is C1. White arrows show cartilage thickness in trochlear region of lateral anterior femoral condyle.

**Figure 4:**
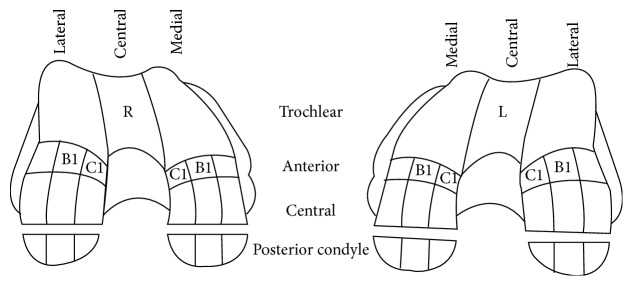
ICRS alphanumerical mapping of cartilage lesions. B1 and C1 region of the anteromedial femoral condyle of both knees are indicated in the images. (Adapted from ICRS Cartilage Injury Evaluation Package.)

Two independent reviewers looked specifically for FNMFH on FIESTA images that is adherent to the anterocentral (B1) and anteromedial (C1) regions of the medial femoral condyle (MFC) and lateral femoral condyle (LFC) (Figures 1–3). The articular cartilage of the outer third regions of the MFC and LFC (A) is incomplete anteriorly (Figure 1). The middle third regions of the MFC and LFC (B) have cartilage in all sections (Figure 2). The articular cartilage of the inner third regions of the MFC and LFC (C) is incomplete posteriorly (Figure 3). The correlation of the above mapping system to the known ICRS system of arthroscopic mapping was taught to the surgeon (Figure 4) [11].

The grade of cartilage abnormality was assessed using the Modified Outerbridge Classification [12]. There are five grades of cartilage abnormalities. Grade 0 refers to normal cartilage; grade 1 is abnormal intrachondral signal (signal increase in T2 weighted images) but normal chondral surface cartilage; grade 2 is loss of less than 50% of cartilage but without exposure of subchondral bone defect; grade 3 is loss of more than 50% of cartilage thickness but without exposure of subchondral bone; grade 4 is complete loss of cartilage with subchondral bone exposure. Cartilage abnormality had to be present in at least two consecutive slices under the FNMHF. The cartilage was considered to be normal if the band of intermediate signal intensity had a uniform thickness. Each patient's registration number, sex, and age are recorded. The age and sex of patients are used to find the prevalence of cartilage abnormalities among patients of different ages and sexes. The MRIs were then independently reviewed by 2 reviewers and categorized into the 5 above-mentioned categories.

We followed up patients with the FNMHF adjacent to the B1 and C1 on MRI to find out those who went on to arthroscopy. For three of the study patients who went for arthroscopy we asked the orthopaedic surgeon who is involved in this study to take images of the chondral defects and biopsy of FNMHF was taken. The synovial biopsy samples were examined using the haematoxylin and eosin staining protocol.

### 2.5. Statistical Analysis

Data obtained were keyed into a Microsoft Excel spreadsheet (Redmond, WA). Statistical analysis was done using the SPSS (IBM, USA) version 20.0.

Mann-Whitney *U* test was used to look for differences in ages between groups with versus without FNMHF and also between groups with versus without cartilage abnormality. Chi-square test was used to test the null hypothesis that there is a significant association between FNMHF and cartilage abnormality. *p* values of less than 0.05 were taken to be statistically significant. The sensitivity, specificity, accuracy, and 95% confidence intervals were calculated. Kappa statistics were used to test interrater agreement for the presence of synovitis and cartilage abnormality.

## 3. Results

One hundred and forty-eight subjects (149 knees, 596 (149 B1 MFC, 149 C1 MFC, 149 B1 LFC, and 149 C1 LFC) subregions) were included. The mean age of subjects was 30 years (range 13–50 years, ±8.90). Of the 148 subjects, 32.8% were women (*n* = 49). There was a significant association found between FNMHF and cartilage abnormality (*p* < 0.01). Considering all four compartments together, there were no significant differences between the groups with versus without FNMHF found for age (*p* = 0.56). There were also no significant differences between the groups with versus without cartilage abnormality found for age (*p* = 0.25).

The prevalence of cartilage abnormality in our study population was 32.0% (*n* = 47). The presence of FNMHF can predict the presence of a cartilage abnormality in the adjacent femoral condyle with a sensitivity of 78.4% and specificity of 97.8%. Good interrater agreement was achieved for the presence of FNMHF and cartilage abnormality (*K* = 0.97 for both). The positive and negative predictive values obtained were 70.7% and 98.5%, respectively.

**Figure 5:**
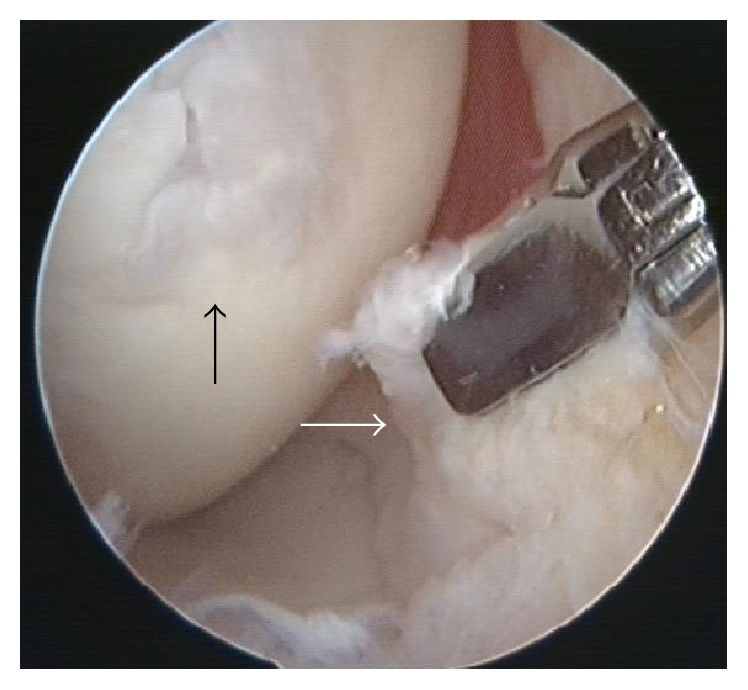
Focal nodular reactive changes adjacent to the focal cartilage defect (black arrow) in the absence of generalized synovitis (*white arrow*).

**Figure 6:**
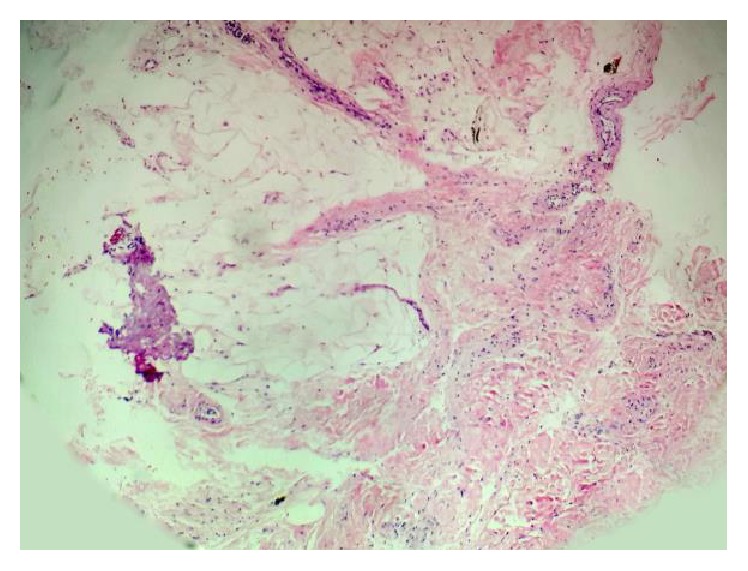
Histology of synovial biopsy. Section shows fibrocollagenous and fatty tissue containing blood vessels. No inflammation seen.

Arthroscopy in this patient revealed focal nodular reactive changes adjacent to the focal cartilage defect in the absence of generalized synovitis (Figure 5). The synovial biopsy samples which were useful to the pathologist revealed fibrocollagenous and fatty tissue with no inflammatory cells (Figure 6).

## 4. Discussion

Normal synovium is the most vascular portion of the diarthrodial joint and is the first mediator of a disease process. Conditions that cause irritation of the intraarticular structures will cause an inflammatory response within the synovium [13].

Our study is novel as we are trying to demonstrate the protective effects of the normal synovial membrane on the articular cartilage of the knee on MRI. Most studies so far have investigated the role of synovitis in the progression of osteoarthritis [14, 15]. Our approach was to evaluate MRI of young and active adults who did not have clinical or radiological signs of osteoarthritis.

Our null hypothesis states that there is no association between FNMHF and adjacent cartilage abnormalities. Our study found that 85% of articular cartilage injury is associated with adjacent reactive FNMHF demonstrating the close relationship between these two integral structures of the knee. More importantly, our data showed that FNMHF has very high negative predictive value for chondral injury (97.8%). The absence of FNMHF contacting the articular cartilage would indicate a very low possibility of a concomitant chondral lesion. This is an indirect evidence that supports the suggestion that FNMHF occurred in conjunction with chondral injury. This has been postulated to be a form of secondary reactive synovitis thought to play a role in the repair of damaged cartilage [16]. Only 5 patients had isolated FNMHF without cartilage abnormality. Ideally, arthroscopy could have been done for these patients to ascertain the presence of any subtle chondral defects.

Our arthroscopic finding agrees with that of Ayral et al. [17] and Kurosaka et al. [18]. The villi were not significantly different from those of normal synovium, retaining their slender, thin, and membranous appearance. The vascular pattern tends to be normal [18]. Biopsy of the FNMHF revealed fibrocollagenous and fatty tissue containing blood vessels with no inflammation seen and this is an important finding as it may imply the provision of healing fibrocollagen to heal the underlying cartilage defect.

**Figure 7:**
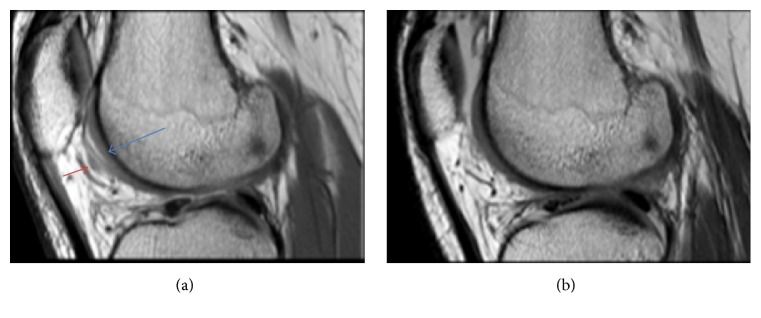
(a) Sagittal FIESTA image showing a grade 2 cartilage defect (blue arrow) with FNMHF (red arrow). (b) Follow-up MRI 2 years later showing healed cartilage defect and absent synovial thickening.

Most of our patients did not have follow-up MRIs which is one of the limitations of our study. However, one of the subjects had a follow-up MRI 2 years after the initial scan. The subject was a 29-year-old male with a history of previous sports injury. He sustained lateral meniscus, anterior cruciate ligament, and medial collateral ligament injuries as well as grade 2 cartilage injury to the C1 region of the lateral femoral condyle injuries. The initial MRI showed FNMHF and a grade 2 cartilage abnormality in the C1 region of the LFC (Figure 7(a)). He did not receive any intra-articular or systemic corticosteroids treatment and the follow-up MRI showed that his chondral injury had healed and the focal area of synovial thickening had also vanished (Figure 7(b)). This suggests that the FNMHF seen on the first MRI was most likely normal healing synovial proliferation.

This differs from the synovial thickening found in osteoarthritic knees. Although the general consensus on the histological appearance of synovitis in osteoarthritis and secondary reactive synovial proliferation is that it is nonspecific with overlapping features, in a study of 9 patients with mild osteoarthritis, synovial biopsies in all patients showed mild chronic synovitis. There was modest degree of papillary hyperplasia of synovial membrane, a mononuclear cell infiltrate, and proliferation of small blood vessels [19]. Thus, even in the absence of synovial thickening on MRI, our synovial biopsy proves there is normal synovial proliferation without evidence of inflammation which may suggest an ongoing healing process.

We can postulate that the FNMHF behaves in a similar fashion to the omentum, plugging the leak in the hollow viscus, mending the chondral injury. This has paramount importance as arthroscopists have a tendency to scrape off any excess synovial proliferation, believing that it not only has nothing to do with healing but also contributes to possible impingement.

The potential for cartilage repair seems to be better in younger individuals and it has been stated that the ideal patient for cartilage repair surgery is younger than 45 years old and has asymptomatic isolated chondral defect with no evidence of osteoarthritis [20]. All but two of our study patients fit this profile. One patient had two chondral lesions and the other was 47 years of age. Therefore, it would be reasonable to suggest that should these and other young patients with small isolated chondral lesions undergo arthroscopy, any reactive synovitis encountered should not be shaved off as this may impair the healing process. One further area of study which may be important is whether patients with FNMHF with cartilage abnormality may be more likely to benefit from mesenchymal stem cell or other regenerative injections into the joint as the stem cells and regenerative agents may incorporate into the FNMHF and assist the healing process.

## 5. Limitation

There were several limitations to our study. Initially, we had 383 patients. However, many subregions were excluded because they were not assessable, mainly because of motion artefacts or field inhomogeneity at baseline and/or follow-up, which did not allow scoring of the features in these subregions.

We did not take into consideration the duration of any existing symptoms and the time interval between onset and MRI examination. Further studies are needed to investigate the effect of treatment on the evolution of synovial proliferation and to substantiate the role of synovium in the repairs of chondral lesion.

Another limitation of our study was the lack of systematic surgical correlation. Such correlation is ideally obtained with arthroscopy, in which the articular compartments are assessed thoroughly. Use of this technique is not universally accepted, however, and differs greatly among orthopaedic surgeons [6]. Furthermore, it is not ethically acceptable to perform an arthroscopy however minuscule the complications are, particularly when imaging findings are normal.

Even though we could prove an association between synovitis and cartilage injury of the knee, we cannot be sure of the chronological order of these features. During the course of our study, we looked specifically at the anterior femoral condyle region as this region provided the largest area of synovial covering and any thickening in this region would be easily appreciated.

We excluded the weight bearing subregions such as B2 and C2 regions in the medial and lateral femoral condyles which we noticed also had cartilage abnormalities. One could argue that this may have introduced bias.

We did not have histological proof of secondary reactive synovitis in most cases, because either there was no indication for arthroscopy or arthroscopy was declined by the patient. Perhaps if the study was carried out over an extended period of time, these patients could have been followed up to assess the resolution or progression of these cartilage defects on MRI.

## 6. Conclusion

Our study showed that routine MRI pulse sequences are useful in identifying the presence of FNMFH which may assist to locate cartilage defects in the knee. As we know surgeons today are more aggressive in management of grade 2 to grade 4 cartilage defects so locating these cartilage defects is important. In cases where arthroscopy is performed and FNMFH is noted, it would be advisable that surgeons should probably refrain from shaving off this reactive process which is likely to be a healing process. Further study of FNMFH in relation to its role in assisting injectables such as PRP and mesenchymal stem cells in the healing process by forming natural scaffolding should be conducted in the future.
